# The NeST (Neoadjuvant systemic therapy in breast cancer) study: National Practice Questionnaire of United Kingdom multi-disciplinary decision making

**DOI:** 10.1186/s12885-020-07757-6

**Published:** 2021-01-22

**Authors:** I. Whitehead, G. W. Irwin, F. Bannon, C. E. Coles, E. Copson, R. I. Cutress, R. V. Dave, M. D. Gardiner, M. Grayson, C. Holcombe, S. Irshad, C. O’Brien, R. L. O’Connell, C. Palmieri, A. M. Shaaban, N. Sharma, J. K. Singh, S. Potter, S. A. McIntosh, on behalf of the NeST Study Research Collaborative, Liz Clayton, Ellen Copson, Karina Cox, Tim Crook, Beatrix Elsberger, Ahmed Ghoneima, Sirwan Hadad, Anita Hargreaves, Paul Healy, Adam Heetun, Dan Henderson, Julia Henderson, Natalie Hirst, Fiona Hoar, Mike Hughes, Emma Iddles, Sheeba Irshad, Tracey Irvine, Stacey Jones, Sarantos Kaptanis, Emma MacInnes, Andrew McCanny, Linda McLaughlin, Anthony Neal, Rachel O’Connell, Neill Patani, Belinda Pearce, Mandana Pennick, Simon Pilgrim, Rene Roux, Matthew Rowland, Sunita Saha, Kavita Sharma, Jagdeep Singh, Chiara Sirianni, Brendan Skelly, Rachel Soulsby, Mark Tatterton, Rob Thomas, Medy Tsalic, Raghavan Vidya, Olivia Waker, Lisa Whisker, Ian Whitehead, Janet Woods

**Affiliations:** 1 Royal Liverpool University Hospital, Liverpool University Hospitals NHS Foundation Trust, Prescot Street, Liverpool, L7 8XP UK; 2 Belfast Health and Social Care Trust, Belfast City Hospital, Lisburn Road, Belfast, BT9 7AB UK; 3 Centre for Public Health, Queen’s University Belfast, Institute of Clinical Science, Block A, Royal Victoria Hospital, Belfast, BT12 6BA UK; 4 University of Cambridge, Cambridge, UK; 5 Cancer Sciences Academic Unit, Faculty of Medicine, University of Southampton, Southampton, SO16 6YD UK; 6 The Nightingale Centre, Wythenshawe Hospital, Manchester University NHS Foundation Trust, Manchester, M23 9LT UK; 7 Department of Plastic Surgery, Wexham Park Hospital, Frimley Health NHS Foundation Trust, Slough, SL2 4HL UK; 8 NI Cancer Research Consumer Forum, c/o NI Cancer Trials Network, East Podium, C-Floor, Belfast City Hospital, Belfast, BT9 7AB UK; 9 Liverpool University Hospitals Foundation Trust, Prescot Street, Liverpool, L7 8XP UK; 10 Guy’s Cancer Centre, Guy’s & St Thomas’ NHS Trust, Great Maze Pond, London, SE1 9RT UK; 11 School of Cancer & Pharmaceutical Sciences, King’s College London, London, SE1 9RT UK; 12 The Christie Hospital NHS Foundation Trust, Wilmslow Road, Manchester, M20 2BX UK; 13 School of Medical Sciences Faculty of Biology, Medicine and Health University of Manchester, Manchester, M13 9PL UK; 14 Royal Marsden NHS Foundation Trust, Downs Road, Sutton, Surrey, SM2 5PT UK; 15 University of Liverpool, Institute of Systems, Molecular and Integrative Biology, Department of Molecular and Clinical Cancer Medicine, Liverpool, UK; 16 The Clatterbridge Cancer Centre NHS Foundation Trust, Liverpool, UK; 17 Queen Elizabeth Hospital Birmingham and University of Birmingham, Mindelsohn Way, Edgbaston, Birmingham, B15 2GW UK; 18 Breast Unit, Level 1 Chancellor wing, St James Hospital, Beckett Street, Leeds, LS97TF UK; 19 Bristol Centre for Surgical Research, Population Health Sciences, Bristol Medical School, Canynge Hall, 39 Whatley Road, Clifton, Bristol, BS8 2PS UK; 20 Bristol Breast Care Centre, North Bristol NHS Trust, Southmead Hospital, Southmead Road, Bristol, BS10 5NB UK; 21 Patrick G Johnston Centre for Cancer Research, Queen’s University Belfast, 97 Lisburn Road, Belfast, BT9 7AE UK

**Keywords:** Breast cancer, Neoadjuvant treatment, Chemotherapy, Endocrine therapy, Surgery

## Abstract

**Background:**

Neoadjuvant systemic therapy (NST) is increasingly used in the treatment of breast cancer, yet it is clear that there is significant geographical variation in its use in the UK. This study aimed to examine stated practice across UK breast units, in terms of indications for use, radiological monitoring, pathological reporting of treatment response, and post-treatment surgical management.

**Methods:**

Multidisciplinary teams (MDTs) from all UK breast units were invited to participate in the NeST study. A detailed questionnaire assessing current stated practice was distributed to all participating units in December 2017 and data collated securely usingREDCap. Descriptive statistics were calculated for each questionnaire item.

**Results:**

Thirty-nine MDTs from a diverse range of hospitals responded. All MDTs routinely offered neoadjuvant chemotherapy (NACT) to a median of 10% (range 5–60%) of patients. Neoadjuvant endocrine therapy (NET) was offered to a median of 4% (range 0–25%) of patients by 66% of MDTs. The principal indication given for use of neoadjuvant therapy was for surgical downstaging. There was no consensus on methods of radiological monitoring of response, and a wide variety of pathological reporting systems were used to assess tumour response. Twenty-five percent of centres reported resecting the original tumour footprint, irrespective of clinical/radiological response. Radiologically negative axillae at diagnosis routinely had post-NACT or post-NET sentinel lymph node biopsy (SLNB) in 73.0 and 84% of centres respectively, whereas 16% performed SLNB pre-NACT. Positive axillae at diagnosis would receive axillary node clearance at 60% of centres, regardless of response to NACT.

**Discussion:**

There is wide variation in the stated use of neoadjuvant systemic therapy across the UK, with general low usage of NET. Surgical downstaging remains the most common indication of the use of NAC, although not all centres leverage the benefits of NAC for de-escalating surgery to the breast and/or axilla. There is a need for agreed multidisciplinary guidance for optimising selection and management of patients for NST. These findings will be corroborated in phase II of the NeST study which is a national collaborative prospective audit of NST utilisation and clinical outcomes.

## Background

Breast cancer affects around 55,000 women per year in the United Kingdom, and over recent years breast cancer management has evolved, with increasing use of personalised approaches to therapy. This includes the use of neoadjuvant systemic therapy (NST), which may be used to reduce the extent of surgery, as well as to determine the sensitivity of a tumour to therapy in the in vivo setting. Long-term results from randomised control trials comparing neoadjuvant with adjuvant chemotherapy (NACT) have demonstrated no significant difference in distant recurrence, breast cancer mortality or any cause mortality [1]. Furthermore, pathological response to neoadjuvant chemotherapy has been validated as predictive of long-term outcomes, and meta-analysis has shown that approximately ~ 26% of unselected patients can achieve a pathological complete response (pCR) [2, 3]. Pathological complete response (pCR) is higher in some disease subtypes, with for example HER2-positive breast cancers achieving pCR rates of up to 60% [4], with much lower response rates reported in ER-positive disease [2]. The presence of residual disease following neoadjuvant therapy may inform the choice of subsequent adjuvant therapies, with consequent improved outcomes [5, 6]. Furthermore, national guidance recommends offering primary systemic therapy to ER negative and HER2 positive invasive breast cancer, with international guidance recommending this approach for the treatment of stage 2 or 3 HER2 positive or triple negative disease [7, 8].

Primary endocrine therapy has long been used in the management of hormone receptor positive patients deemed unfit for surgery, although the role of neoadjuvant endocrine therapy (NET) is still being established. Some studies have demonstrated it to be as effective as chemotherapy in strongly hormone receptor positive disease, and the Pre-operative Endocrine Prognostic Index has been reported as a tool for use following NET to identify patients at risk of relapse [9]. The majority of trials in this setting are low powered with small sample sizes and variable treatment duration, and the extent of NET use within UK centres remains unclear [10–12].

In both Europe and the United States, significant regional variation has been reported in the use of neoadjuvant chemotherapy [13, 14]. Similar variation has been demonstrated in the UK, in the national Mastectomy Decisions Audit (MasDA) [15]. This national prospective study showed that many centres opted for mastectomy over NST in almost 30% of patients with large tumour to breast size ratio, missing potential opportunities for surgical downstaging. Indeed, in HER2 positive disease some 60% of patients potentially eligible for neoadjuvant therapy proceeded directly to mastectomy without such treatment in this study.

Furthermore, it has been shown that even where improved pCR rates can be achieved this does not necessarily impact on rates of breast conserving surgery [16]. This may be due to differences in the surgical approach to the breast following neoadjuvant therapy, with some surgeons resecting the original tumour footprint irrespective of response to treatment, whilst others will tailor their resection to residual disease. Timing of axillary staging and surgical approach to patients presenting with node-positive disease is also controversial [17]. Finally, there is little consensus in the imaging modalities used to monitor treatment response in the neoadjuvant setting, nor in pathology reporting frameworks across the UK.

This study aimed to describe the current stated practice of multidisciplinary teams (MDTs) across the UK with respect to the use of neoadjuvant systemic therapy, including indications for use, response monitoring, pathological reporting and surgical approaches to the breast and axilla. It forms the first stage of the Neoadjuvant Systemic Therapy in Breast Cancer (NeST) Study, a national multicentre, multidisciplinary collaborative prospective study assessing usage and real-world outcomes [18].

## Methods

All surgical and oncological units treating breast cancer within the UK were eligible to participate in the NeST study. As with previous National Practice Questionnaires in the UK, multidisciplinary units were invited to participate by email in December 2017 via a number of professional and research organisations [19]. These included the Mammary Fold (MF) Breast Trainees’ Association, the Association of Breast Surgery, the Association of Surgeons in Training, the National Trainee Research Collaborative (NTRC), the Breast Cancer Trainees Research Collaborative Group, the NCRI Breast Clinical Studies Group (CSG) and the Reconstructive Surgery Trials Network (RSTN). Thus each unit received an invitation to participate through several sources during December 2017, with a further reminder invitation through these routes sent in January 2018. No information was collected on units which did not respond to the invitations.

Members of the NeST steering committee developed the national practice questionnaire (NPQ), which was piloted in 4 centres and iteratively modified according to feedback, to ensure ease of use. The final 68 item questionnaire collected data on multidisciplinary team (MDT) demographics, indications for NST, proportion of patients offered NST, preferred treatment regimes, methods of monitoring response, subsequent surgical management and reporting of pathological response. The questionnaire was issued to all MDTs participating in the NeST study via the secure electronic database, REDCap in December 2017 [20], and is provided as Supplementary Data. Respondents were asked to complete the questionnaire at their weekly multidisciplinary meetings, where all specialties were present. Data was uploaded to REDCap by the local lead for the NeST study. MDTs with incomplete NPQs were contacted via e-mail and invited to input missing data in order to maximise data capture, which was completed by September 2019.

All participating institutions gained local governance approval for participation in the study.

Simple summary statistics were calculated for each questionnaire item. Categorical data were summarised by counts and percentages. Continuous data was summarised by mean, median, standard deviation and ranges as appropriate. Statistical tests were carried out using Graphpad Prism 9.

## Results

Responses were obtained from a total of 39 of 144 UK MDTs. Characteristics of participating centres’ service provision and MDT composition are summarised in Table 1.

**Table 1:** Demographics of participating breast units and multidisciplinary teams

Organisation	Number (%)
Teaching Hospital	23 (59)
DGH	15 (38)
Not stated	1 (3)
**Service Provision**	
Symptomatic only	3 (8)
Screening/symptomatic	36 (92)
**Actively recruiting to trials**	
	100 (39)
**Unit size**	**Median cases per year (range)**
	470 (220–1000)
**MDT Composition**	**Median No. consultants (range)**
Histopathologist	2 (1–8)
Radiologist	4 (1–9)
Oncologists	4 (1–10)
Clinical oncologist	2 (0–5)
Medical oncologist	2 (0–5)
Breast Surgeons	3 (0.5–10)
Oncoplastic Breast Surgeon	3 (0.5–10)

### Indications and selection for Neoadjuvant therapy

All MDTs in the study reported routinely offering NACT to their patients, with an estimated median of 10% (range 5–60%) cases being offered this modality. The median usage of NACT in teaching hospitals was 10% (range 5–40%), as compared with 7.75% in DGHs (range 5–60%) (*p*=0.32, Mann-Whitney U test).

Twenty–six MDTs (66% of the total) routinely offered NET; a further 5% (*n*=2) offer NET only as a treatment option within a clinical trial. For teaching hospitals, 22% did not routinely offer NET, whereas for DGHs this figure was 27%. A median of 4% (range 0–25%) of patients were offered NET at these centres, with a median duration of treatment of 6 months (range: 3–9 months). At teaching hospitals, the median number of patients offered NET was 5% (range 0.2–25%), and at DGHs the corresponding figures were 2.5% (range 0.5–12.5%) (*p*=0.41, Mann-Whitney U test).

**Fig. 1:**
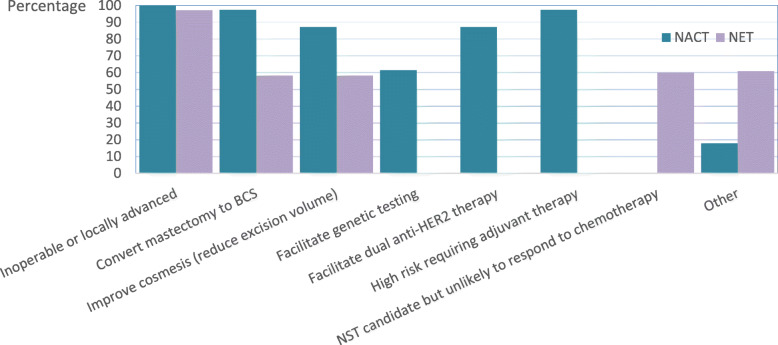
Histogram depicting stated indications for use of neoadjuvant chemotherapy and endocrine therapy. If selecting “Other” then respondents were invited to provide a free text response. Other responses were “downstaging of heavy nodal disease”, “HER2+ve disease ≥2 cm in diameter”, and “to allow patients time to prepare psychologically for mastectomy/reconstruction”

Indications for recommending neoadjuvant therapy are summarised in Fig. 1. The most common indication of the use of both NACT and NET was for the downstaging of disease, either to treat locally advanced disease or to downstage planned surgery.

**Fig. 2:**
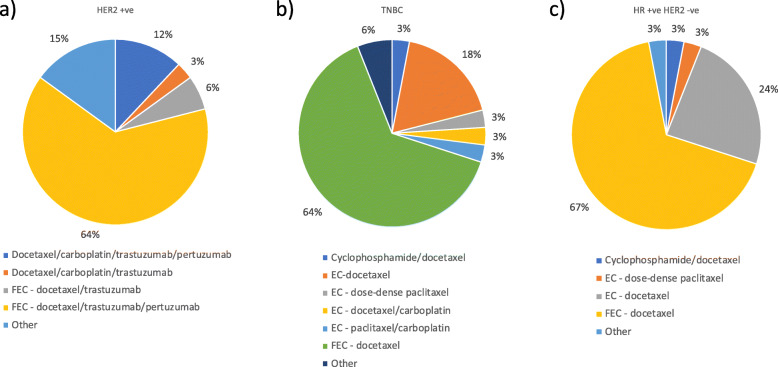
Most commonly prescribed neoadjuvant chemotherapy regimes according to disease subtype. **a** HER2 positive cancer **b** Triple negative breast cancer (TNBC) **c** Hormone receptor positive, HER2 negative

Neoadjuvant chemotherapy regimens most commonly reported being used are summarised in Fig. 2, with the most commonly prescribed regimen being FEC-docetaxel/trastuzumab/pertuzumab for HER2-positive disease and FEC-docetaxel for HER2-negative disease. Preferred regimens were not stated in the responses from 6 MDTs (15%).

Neoadjuvant radiotherapy use in the UK is low, with 58% of respondents reporting that they do not use this approach, and a further 38% stating that they would only use it in the context of advanced or inoperable disease unresponsive to systemic therapies and one unit only using it in the context of a clinical trial (3%). One unit did not respond to this question.

### Monitoring and management of treatment response

Monitoring of response information was provided for all MDTs. A marker clip is routinely sited in the breast by 97% of multidisciplinary teams when using NACT. In 79% of units this is prior to commencing treatment, with 21% varying the timing due to practicalities.

**Fig. 3:**
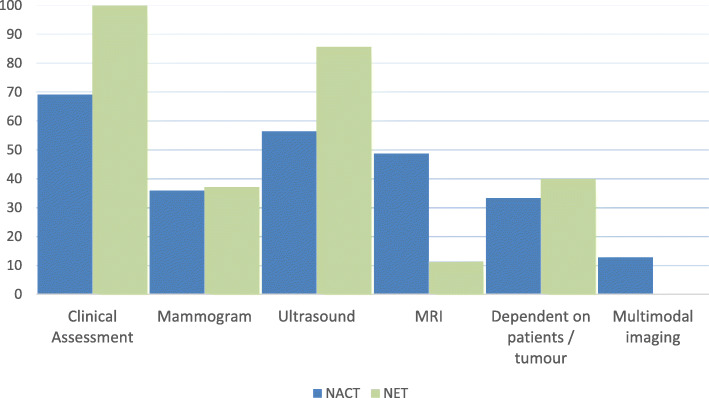
Prefered modalities used in the monitoring of neoadjuvant systemic therapy response. Represented as percentage of MDTs using each modality for Fig. 3 **a**: neoadjuvant chemotherapy and Fig. 3 **b** neoadjuvant endocrine therapy. As some units use more than one imaging modality, percentages may add up to > 100%

Preferred modalities for monitoring response to treatment are detailed in Fig. 3 (a) and (b) for neoadjuvant chemotherapy and endocrine therapy respectively, with clinical assessment and/or ultrasound being utilised most often. Response is assessed at varying time points during treatment as follows:

- 45% mid-point and end of treatment
- 5% mid-point, end and other time point
- 3% mid-point and other time point
- 18% mid-point only
- 8% end of treatment only
- 18% other time point only
- 3% - varies with MDT consideration of cancer and patient characteristics

A quarter of MDTs (27%) stated they do not monitor response in patients planned to undergo mastectomy. Where response to NACT is monitored, results are routinely discussed in MDT meetings at 76% of centres with 22% of centres discussing selected patients only, and 2% (one centre) foregoing MDT discussion completely.

When using NET, 75% of MDTs site a marker clip; 86% of these centres deploy the clip prior to treatment, with 7% siting it during treatment and 7% varying the timing in response to practicalities. The median reported duration of NET was 6 months (range 3–9 months) before proceeding to surgery. All centres using NET monitor response clinically, with 95% also using radiological modalities (Fig. 3). Patients on NET are routinely discussed in 60% of MDTs, selectively discussed in 30% of MDTs and not discussed in 10% of MDTs.

### Post NST loco-regional treatment

When managing the breast post NST 74% of centres practise response-adapted surgery whereas 26% stated that they resect the original tumour footprint, regardless of the extent of clinical or radiological response to treatment. The majority of centres carry out post-NST sentinel lymph node biopsy (SLNB) in patients with clinically negative axillae at diagnosis (73% post-NACT and 84% post-NET). In patients with clinically positive axillary nodes at diagnosis 60% of centres stated that they would carry out axillary node clearance (ANC) regardless of response to NACT, and 69% (*n*=25) following NET. Thirteen percent of MDTs would re-assess the axilla following NACT and 25% following NET prior to making a surgical decision.

Post-NST, virtually all units stated that they would treate the conserved breast with adjuvant radiotherapy (97%). Post-mastectomy radiotherapy (PMRT) was largely driven by pre-treatment tumour size and nodal status, with 92% of MDTs stating that they give PMRT where pre-treatment tumour size was ≥50 mm, and 87% giving supraclavicular fossa (SCF) radiotherapy based on a pre-treatment diagnosis of N2 disease.

Thirty six percent of units that perform SLNB prior to NST would proceed to an ANC post-treatment, without further assessing the axilla if sentinel nodes are positive. Patients found to have a positive axilla on post-NST SLNB are managed on an individualised basis at 31% (*n*=11) of centres following NACT and 54% (*n*=19) of centres following NET. Approximately half of MDTs would perform a completion ANC; 54% (n=19) following NACT, and 46% (*n*=16) for NET.

### Histopathology

**Fig. 4:**
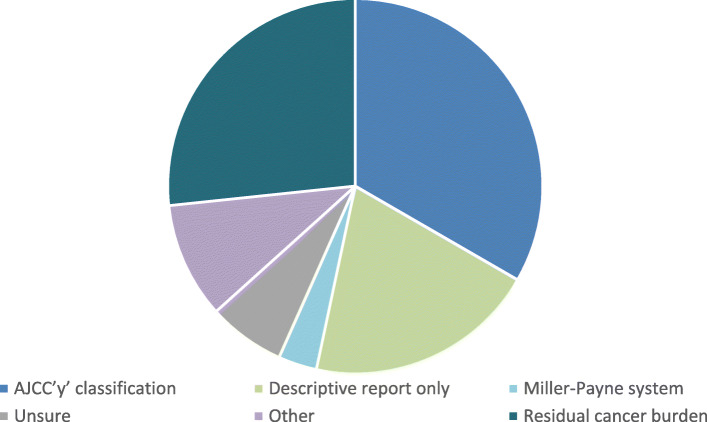
Histopathology reporting systems post neoadjuvant chemotherapy

In 86% of MDTs, a reporting system is routinely used to describe the extent of pathological response to NACT. Figure 4 summarises the reporting systems used, with 2 centres not responding to this question (5%). Ki67 is routinely measured in post-NST specimens in only 11% of centres, with 8% reporting it in selected circumstances such as clinical trials, and 5% not responding. In contrast, only 46% of MDTs use a system to report response to NET, and 14 centres use a descriptive report only.

## Discussion

This study gives an overview of the stated practice in the use of neoadjuvant systemic therapy for breast cancer in the UK, confirming that wide variation in such practice exists. All 39 participating centres in the first phase of the NeST Study offer NACT, although it appears that a relatively small proportion of patients (median 10%, range 5–60%) are recommended this treatment strategy. There is wide variation reported in the frequency with which NACT is recommended in UK MDTs, although we showed no difference in stated rates of use between teaching hospitals and DGHs, albeit in a relatively small number of hospitals using self-reported data. When considering the findings of studies such as MasDA, and considering the potential benefits for patients of using NST (both in terms of surgical downstaging and the utility of treatment response as a prognostic biomarker), it is likely that there is under-utilisation of this approach across the UK [15]. Furthermore, the variation in usage of NACT between MDTs appears too wide to be accounted for by variation in patient populations between units, although it is known that the age structure of the UK population varies by local area, and that use of both NACT and NET may consequently vary with age [21]. Individual patient-level data for patients being treated with NeST was not collected in the National Practice Questionnaire; however, the prospective audit phase of NeST will seek to explore this aspect of neoadjuvant therapy usage in more detail [18].

It remains clear that surgical downstaging is a primary indication for the use of NACT in many MDTs. However, it is equally clear that other indications for recommending NACT are emerging, in line with disease biology. Response to treatment has been shown to be a valuable predictor of long-term outcome following neoadjuvant chemotherapy [22]. However, it is also increasingly clear that pathological response to treatment can be utilised as a functional biomarker, to guide the use of subsequent adjuvant therapies where patients have an incomplete response to treatment, particularly in the context of certain disease subtypes, such as HER2+ or triple negative disease [5, 6].

Around two thirds of MDTs in this survey are using NET. However, this approach tends to be offered only to a small proportion of patients (a median of 4% in this study), and appears to be primarily used in the UK where disease is considered to be locally advanced or inoperable, to facilitate surgical treatment, with a relatively small proportion of centres employing this approach to downstage disease to reduce the extent of surgery. Similarly low usage of NET was seen in the MasDA study, although many post-menopausal women with ER-positive breast cancer were recommended mastectomy due to a large tumour to breast size ratio, and could potentially have benefitted from NET [15]. A 2016 metanalysis suggests that NET with aromatase inhibitors is comparable to NACT in terms of radiological and clinical response rates, with similar rates of breast conserving surgery, [11]. In this meta-analysis it is noted that 90% of published studies of NET include post-menopausal women, and that there is little data on the use of NET in pre-menopausal women with hormone receptor positive breast cancer. Clearly the role and benefits of NET in such pre-menopausal women is yet to be established, and the prospective audit phase of NET will determine the age distribution and menopausal status of women in the UK being treatment with NET [18]. However, this data, taken together with the MasDA findings show that there remains a clear reluctance to utilise this approach routinely in clinical practice within the UK despite the low pCR reported with NACT in this group. The reasons for this remain unclear, but may relate to a perceived lack of evidence regarding the long-term oncological outcomes of this approach. While genomic assays may be of value here and increase clinician confidence in decision-making, there remains a need for further high-quality clinical trial data to guide the management of this patient group.

It is also clear from this data that there is a wide variation in radiological monitoring and pathological reporting during and after neoadjuvant therapy, with no consensus on the optimal radiological method of monitoring response. With respect to pathological reporting, several reporting systems are available, and current UK pathology guidelines do not recommend a particular system [23]. Although the Residual Cancer Burden system is increasingly regarded as the gold standard for reporting pathological response following neoadjuvant chemotherapy and is the system recommended for neoadjuvant trials, only around one quarter of units are using this reporting system routinely, with around 20% of units issuing descriptive reports only [24].

Although surgical downstaging was noted to be a key indication for recommending NAC in this study, around 25% of centres stated that following treatment, the surgical goal remained removal of the original tumour footprint, regardless of response. This is in contrast to the St Gallen consensus guidance, which recommended that excision of the initial tumour bed was not required [25]. It seems likely, therefore, that the opportunity to de-escalate breast surgery following NACT is not being fully utilised in some patients.

Management of the axilla following NACT has been a controversial area, although subsequent to this survey, UK multidisciplinary recommendations have been produced to guide treatment [26]. At the time of this study, the majority of patients diagnosed with clinically node negative breast cancer receiving NACT were undergoing post-treatment SLNB. However, in patients presenting with node-positive disease, the majority of centres were performing axillary node clearance, with only a small number of centres carrying out axillary reassessment and response-guided treatment of the axilla. It appears likely, therefore, that a proportion of patients may not have the opportunity for de-escalation of axillary surgery following NACT, although the impact of the published guidance remains to be seen.

Clearly, there are some limitations to the data provided by this questionnaire. Only 39 of 144 breast units (27%) in the UK participated in the study, and consequently there may be selection bias as these units may not be representative of UK practice more broadly. Although this data has been supplied by all members of the MDT and therefore should represent an accurate reflection of multidisciplinary perspectives, we accept that this study is based on reported rather than actual practice of MDTs. We note that the self-reported range of NACT use is from 5 to 60%, with the upper limit appearing surpriginly high. While this may reflect genuine variation in NACT use (and that some centres, including specialist tertiary referral centres are high users of this treatment approach), it is possible that self-reported data may not be entirely reflective of actual patterns of care. Consequently the data may be reflective of perceived rather than actual practice, as studies (albeit in other specialties) have demonstrated there to be a difference between these [27]. Furthermore, units were not specifically asked about compliance with national and international guidelines regarding the use of NeST such as UK NICE guidance or the St Gallen guidelines [7, 8]. However, the prospective audit phase of the NeST study will provide valuable insight into decision-making on an individual patient basis across UK MDTs [18].

In spite of these limitations, to our knowledge this is the first UK study to broadly examine real-world stated practice in terms of the use of neoadjuvant systemic therapy. It is clear that there is wide variety in perceived indications for and use of neoadjuvant systemic therapy in the UK, as well as a lack of consensus on the optimal methods for monitoring and reporting response and on the surgical management of the primary tumour following systemic therapy. Although NICE guidance outlines potential indications of the use of NST in breast cancer, this study indicates a clear need for both further research and the development of multidisciplinary guidance with respect to monitoring of response, pathological reporting and surgical decision-making, to ensure optimal outcomes for breast cancer patients treated with neoadjuvant therapies. We await the prospective phase of the NeST study, which will allow the corroboration of these results with the real-world use of neoadjuvant systemic therapy for breast cancer in the UK [18].

## Data Availability

The datasets generated during and/or analysed during the current study (the NeST National Practice Questionnaire) may be made available upon request from Stuart McIntosh (s.mcintosh@qub.ac.uk). Requests for access to the data will be reviewed by the NeST Study Steering Group prior to any data sharing.

## Appendix

**Table 2:** NeST Study Collaborators

Liz	Clayton	Royal Surrey County Hospital
Ellen	Copson	University Hospitals Southampton NHS Trust
Karina	Cox	Maidstone and Tunbridge Wells
Tim	Crook	Royal Surrey County Hospital
Beatrix	Elsberger	Ninewells Hospital Dundee
Ahmed	Ghoneima	Queen Alexandra Hospital Portsmouth
Sirwan	Hadad	Royal Hallamshire Hospital Sheffield
Anita	Hargreaves	Warrington & Halton NHS Trust
Paul	Healy	St Vincent’s Hospital Dublin
Adam	Heetun	University Hospitals Southampton NHS Trust
Dan	Henderson	Heart of England NHS Trust
Julia	Henderson	Royal Liverpool University Hospital
Natalie	Hirst	Bradford Teaching Hospitals
Fiona	Hoar	City Hospital Birmingham
Mike	Hughes	Airedale General Hospital
Emma	Iddles	Aberdeen Royal Infirmary
Sheeba	Irshad	Guys Kings & St Thomas’
Tracey	Irvine	Royal Surrey County Hospital
Stacey	Jones	St James’ University Hospital Leeds
Sarantos	Kaptanis	Bedford Hospital
Emma	MacInnes	Doncaster Royal Infirmary
Andrew	McCanny	Ulster Hospital Dundonald
Linda	McLaughlin	
Anthony	Neal	Royal Surrey County Hospital
Rachel	O’Connell	Royal Surrey County Hospital
Neill	Patani	Royal Marsden Hospital
Belinda	Pearce	Royal Hampshire County Hospital
Mandana	Pennick	Betsi Cadwaladr - Glan Clywd hospital
Simon	Pilgrim	University Hospitals Leicester
Rene	Roux	Oxford University Hospitals NHS Trust
Matthew	Rowland	Royal Devon & Exeter
Sunita	Saha	Colchester General Hospital
Kavita	Sharma	Glasgow Royal Infirmary
Jagdeep	Singh	University Hospitals Birmingham
Chiara	Sirianni	Betsi Cadwaladr
Brendan	Skelly	Belfast City Hospital
Rachel	Soulsby	Milton Keynes University Hospital
Mark	Tatterton	Salisbury District Hospital
Rob	Thomas	Royal Victoria Infirmary Newcastle
Medy	Tsalic	Heart of England NHS Trust
Raghavan	Vidya	Royal Wolverhampton NHS Trust
Olivia	Waker	Royal Surrey County Hospital
Lisa	Whisker	Nottingham University Hospitals NHS Trust
Ian	Whitehead	St. Helens and Knowsely Teaching Hospitals
Janet	Woods	Heart of England NHS Trust

## Abbreviations

ABS: Association of Breast Surgery of Great Britain and Ireland
ANC: Axillary node clearance.
DGH: District General Hospital.
MasDA: Mastectomy Decisions Audit.
MDT: Multidisciplinary team.
MF: Mammary Fold.
NACT: Neoadjuvant chemotherapy.
NCRI: National Cancer Research Institute.
NeST: Neoadjuvant systemic therapy.
NET: Neoadjuvant endocrine therapy.
NICE: National Institute for Health and Care Excellence.
pCR: Pathological complete response.
RSTN: Reconstructive Surgery Trials Network.
SLNB: Sentinel lymph node biopsy.
